# 96 Building the customer experience in sports services

**DOI:** 10.1093/eurpub/ckae114.046

**Published:** 2024-09-26

**Authors:** Paula Harmokivi-Saloranta, Tuulia Stellberg

**Affiliations:** Haaga-Helia University Of Applied Sciences, Helsinki, Finland; LAB University of Applied Sciences, Lahti, Finland

## Abstract

**Purpose:**

The experience of the service is not a conscious decision. Customer experience is built on the customer’s interpretations and feelings that they experience during customer journey. The customer experience is influenced by different interaction situations, as well as, for example, the physical environment. In sports services, customers and instructors are in various interaction situations during service journey. Each customer experiences the service in their own way, and for this reason, the experiences of sports services vary between customers. The aim of this study was to examine the factors that influence customer experience in sports services.

**Methods:**

The study was carried out using qualitative methods. There were 22 participants in the study who occasionally use or do not use sports services. On average, the participants were 48 years old. The data were collected through written stories during autumn 2023. Participants described thoughts and feelings related to different phases of sports service journey with the help of assistive questions. The data were analyzed using content analysis.

**Results:**

The results showed that safety experience, encounters, and the instructor’s expertise strengthen the customer experience in sports services. The experience of safety was related to the activities of the sports service and the instructor, the content of the group, other participants of the group, and the surrounding environment. When using the sports service, customers experience fear of how their own competence, fitness, and skill fit into the selected group.

**Conclusion:**

This study provided a broader understanding of the construction of customer experience in sports services. It makes visible which are the main elements that strengthen the customer experience in sports services. The results can be used to develop the customer experience of users of sports services and to promote the use of people’s sports services as part of physical activity promotion.

The research has been co-funded by the European Union.

